# Detection of *Toxocara* species larvae in four Iranian free-range broiler farms

**DOI:** 10.1186/s12917-022-03516-w

**Published:** 2022-11-21

**Authors:** Elham Shokri, Ali Haniloo, Mohammad Zibaei, Ali Pezeshki, Kamyar Mansori, Kensuke Taira

**Affiliations:** 1 Department of Parasitology and Mycology, School of Medicine, Zanjan University of Medical Sciences, Zanjan, Iran; 2 Department of Parasitology and Mycology, School of Medicine, Alborz University of Medical Sciences, P.O. Box: 3149779453, Karaj, Iran; 3 Department of Epidemiology and Biostatistics, School of Medicine, Zanjan University of Medical Sciences, Karaj, Iran; 4 Laboratory of Parasitology, School of Veterinary Medicine, Azabu University, Fuchinobe, Sagamihara, Kanagawa Japan

**Keywords:** *Toxocara*, PCR, Tissue, Free-range broiler chickens, Poultry farms

## Abstract

**Background:**

The epidemiology of *Toxocara canis* and *Toxocara cati* in food animals, associated products, and their zoonotic potential are poorly understood. A cross sectional study was designed to determine the prevalence of *Toxocara* spp. larvae from free-range broiler chickens in traditional farms using conventional techniques and molecular method. Eight-hundred tissue samples including liver, gizzard, lungs and heart were collected from 200 chickens belonging to different regions of Zanjan Province, Iran and were processed by conventional and molecular methods.

**Results:**

Out of 800 chicken tissues, 49 samples (6.1%) were positive for nematode larvae. Polymerase chain reaction was performed to identify species-specific of *Toxocara* larvae. The findings showed that 10.5% (21 out of 200) chickens were infected with *Toxocara* species, so that 57.1% (12 out of 21) of the samples were positive for *Toxocara canis* and 42.9% (9 out of 21) of the samples were positive for *Toxocara cati*.

**Conclusion:**

Considering the significant contamination/infection of free-range broiler chickens with *Toxocara* larvae, the consumption of chicken meat and viscera, especially liver and gizzards, can play an important role in the transmission of infection to humans. Prevention and control measures focused on regular deworming of dogs and cats, increasing public awareness of *Toxocara* infection are recommended.

**Supplementary Information:**

The online version contains supplementary material available at 10.1186/s12917-022-03516-w.

## Background

The dog and cat roundworms, *T. canis* and *T. cati* respectively, are a public health concern in Asia, America, and parts of Europe [1]. The consumption of raw or undercooked viscera or meat of economic animals is a potential risk factor for *Toxocara* infection [2]. The infective larvae are released in the small intestine and subsequently penetrate the intestinal wall, enter the circulation and migrate to various organs, where they provoke inflammatory reactions and symptoms. Based on the organs infected, it is clinically classified into visceral larva migrans, ocular toxocariasis, neural larva migrans, and hidden and/or covert toxocariasis [3]. Known paratenic hosts of *Toxocara* include chicken, cattle, pigs, rodents and birds. Larvae can move into these tissues and survive for a long time [4, 5]. It is thought that the larvae of *Toxocara* species in chicken tissue (gizzard, lungs, kidneys, liver, and heart) are the source of infection for humans [6]. The consumption of raw or undercooked viscera or meat of economic animals is a potential risk factor for *Toxocara* infection [7]. It has been studied that in chicken, most *T. canis* larvae migrate to the liver and remain there [8–10], while most of the *T. cati* larvae migrate to and remain in the muscles [4, 11].

Owing to difficulties with conventional protocols for diagnosis, researchers utilize DNA detection methods because of its rapidity and reliability. Polymerase chain reaction was used to identify and confirm helminth parasites by various researchers worldwide to target specific genes [12–14]. Molecular tools can then provide the level of discrimination that is often not achieved by microscopy alone and which is needed to distinguish between species or intraspecific variants of the relevant parasites. Molecular characterization may provide important information about potential zoonotic risk and transmission dynamics of parasites in a human community.

Molecular epidemiological studies involving animal reservoirs are crucial for determining the sources of helminthic infection in an ecological niche, inferring pathogenic characteristics of species, and developing effective control strategies. It is the most promising method for the epidemiological studies of parasitic infection such as toxocariasis. Molecular epidemiological data on *Toxocara*-infected raw meat used for human consumption in Iran is limited, and to our knowledge, this is the first study to suggest the prevalence of toxocariasis in free-range broiler chickens. Therefore, this study was conducted to investigate the prevalence and identification of *Toxocara* species larvae in four Iranian free-range broiler traditional farms in Zanjan Province in the northwest of Iran.

## Methods

### Ethical consideration

The tissue samples in this study were collected from chickens slaughtered in the traditional markets with the approval of the local animal center of disease control. Animal experiments were not conducted. Therefore, an animal rights statement is not applicable. Permission to conduct this study was also given by the Research Ethics Committee of the Zanjan University of Medical Sciences (IR.ZUMS.REC.1400.272).

### Study area

This study was conducted in Zanjan Province in the northwest of Iran. The province of Zanjan is situated in the northwest of Iran at latitude 36°40′24″ N and longitude 48°28′43″ E. Agriculture and animal husbandry are the principal occupation. The current population of Zanjan Province is a more than a million people. Poultry and sheep meat are the main sources of the animal-source foods for the inhabitants [15].

### Sample collection and necropsy

This cross-sectional study was conducted from April to October, 2021. In each agricultural zone, a traditional market for the supply of local and free-range chickens was selected. In these traditional markets, free-range broiler chickens are offered and delivered to customers after butchery. The selected centers from each agricultural zone were visited once per week and 50 chickens were selected using a 1 in 4 systematic random sampling method [5]. The chicken carcasses were transferred to a parasitology laboratory for examination. Each carcass was dipped in the container of water for washing. Then, the visceral organs including the liver, lungs, gizzard, and heart were eviscerated, separately. The tissues were fragmented into small pieces of less than 5 mm^3^ with pointed forceps for pepsin digestion.

### Digestion method and larval recovery

The digestion method proposed by Azizi et al. [4] and Taira et al. [9] was used with modification. Briefly, the whole organ was cut into 3 × 5 mm fragments and placed in digestive solution [1 g pepsin (Sigma-Aldrich, USA) and 10 mL HCl 37% (Merck, Germany) in 1000 mL distilled water] under constant stirring with a magnetic stirrer and kept for 2 h at 39 °C to recover the remaining. The sediments were filtered through a system of sieves with 246-μm apertures (60-mesh) and allowed to settle for 40 min at room temperature. After the supernatant was removed, sedimented liquids were poured into tubes and centrifuged for 2 min at 252 g. Each of the sediments was transferred to rectangular plastic Petri dishes (Polystyrene, BIOTEST, Zist-Azmoon, Iran), and the presence of any nematode larvae were monitored under a light microscope (Olympus CX23, Japan).

### DNA extraction

DNA was extracted from each larva recovered from the tissues of chickens using the Tissue Genomic DNA Extraction Mini Kit (Favorgen Biotech, Ping-Tung, Taiwan) according to the manufacturer’s instructions. Briefly, 25 mg of the digested sample was transferred to a 1.5 mL Eppendorf tube, suspended in 200 μL lysis buffer (FATG1 Buffer) and 20 μL of Proteinase K. The solution was mixed in a vortex and incubated for up to 3 hr. at 60 °C to lyse the sample. Then, 200 μL FATG2 buffer was added to the sample, mixed thoroughly by pulse-vortexing, and incubated at 70 °C for 10 minutes. The solution was combined with 200 μL of absolute ethanol (Merck, Germany) and it was mixed thoroughly. The mixture was transferred to the FATG Mini Column (supported by the kit) and centrifuged at full speed (~ 18,000 g) for 1 min, then discarded flow-through. After an additional washing with 750 μL of wash buffer, the samples were treated with 100 μL of elution buffer for 3 min at room temperature and centrifuged at full speed for 2 min to elute DNA. Finally, NanoDrop measured the purified DNA concentration (BECO, Germany).

### Molecular characterization

The specific *T. cati* forward primer JW4 (5′-ACTGTCGAGGATGAGCGTGA-3′) was used together with reverse primer NC2 (5′-TTAGTTTCTTTTCCTCCGCT-3′), to amplify the partial internal transcribed spacer-1 (ITS-1), complete 5.8S and ITS-2 of rDNA as described by previous studies [16, 17]. The forward primer YY1 (5′-CGGTGAGCTATGCTGGTGTG − 3′) which is specific for *T. canis* as previously designed and combined with reverse primer NC2 (5′-TTAGTTTCTTTTCCTCCGCT-3′) was used to amplify partial ITS-2 as well [18]. PCR reaction was performed in Super master mix-2X 12.5 μL, primers (forward and reverse) (10 pmol/ μL) 1 μL, DNA template 5 μL, DW 5.5 μL. The PCR conditions were: 95 °C for 5 min for primary denaturation; 94 °C for 45 s for denaturation; 58 °C for 35 s for annealing; 35 cycles at 72 °C for 35 s for extension; 72 °C for 10 min for the final elongation step.

The electrophoresis of PCR products was performed on 1.5% agarose gel, a 100-bp ladder was used as DNA sizemarker for estimating the size of the amplicons, and then the gels were stained in ethidium bromide for 15 minutes and visualized in a gel document system (UV Transilluminator, QUANTUM SD4–1000, VILBER, France).

### Statistical methods

Data and frequencies of *Toxocara* species were entered into SPSS 20.0 Statistics software and subjected to descriptive statistics to determine their percentages. Mann-Whitney, Kruskal-Wallis, and Chi-square (Fisher’s exact) tests were used to evaluate associations of the variables. Differences in all statistical tests were considered significant at *P* < 0.05.

## Results

### Microscopic examination of tissue samples

Of the 200 chickens examined in the present study, 44 (22.0%) were male, and 156 (78.0%) were female. Their age ranged between 2 and 48 (16.1 ± 8.4) months. The body weight of studied chickens was 150–4000 (1740.5 ± 799.7) grams.

**Fig. 1:**
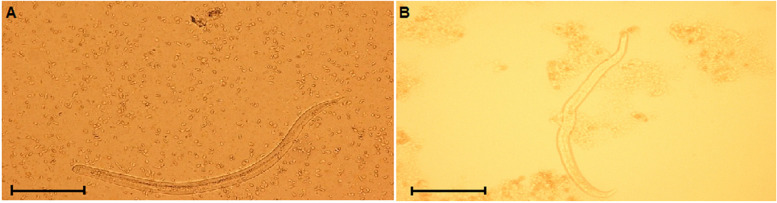
**a** *Toxocara canis* and **b** *Toxocara cati* larvae detected in the liver samples of chickens by artificial enzymatic digestion (Scale bars 100 μm)

In the present study, out of 800 tissue samples examined, 49 samples (6.1%) were positive for the nematode larvae by conventional microscopic examination (Fig. 1). The presences of the larvae were demonstrated in the gizzard (11.5%), liver (9.5%), lungs (2.0%) and heart (1.5%), respectively (Table 1).

**Table 1:** Investigated parameters and recovery of larvae from tissues of free-range broiler chickens by digestion method and microscopic observation

Subject (No.)	Parameters				
Chickens (200)	Age, month	Weight, gram	Female	Male	Infection rate
	Min-max (Mean ± SD)	Min-max (Mean ± SD)	No. (%)	No. (%)	No. (%)
	2–48 (16.1 ± 8.4)	150–4000 (1740.5 ± 799.7)	156 (78.0)	44 (22.0)	33 (16.5)
**Organs (800)**	**Infection rate, No. (%)**				
	Gizzard	Liver	Lungs	Heart	Total
	23 (11.5)	19 (9.5)	4 (2.0)	3 (1.5)	49 (6.1)

### PCR for specific detection

**Fig. 2:**
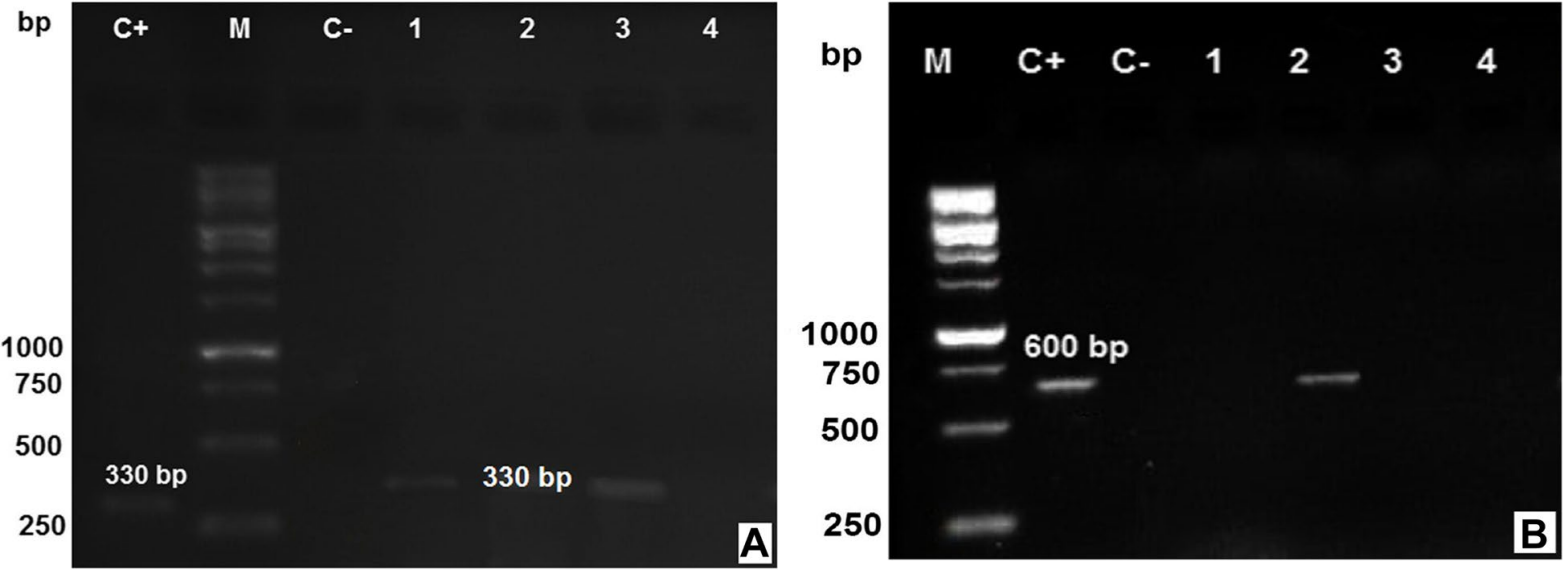
Analysis of PCR products amplified of *Toxocara* species from free-range broiler chicken tissue samples by 1.5% agarose gel electrophoresis. M: Molecular size marker 100-bp, C+: Genomic DNA positive control, and C-: Negative control without DNA. **a** *T. canis* with YY1/NC2 primers, Lanes 1–4: Tissue samples (liver, lungs, heart, and kidney) were collected from the examined chickens. Full-length gel image is provided in Supplementary Fig. 2A. **b** *T. cati* with JW4/NC2 primers, Lanes 1–4: The tissue samples. Full-length gel image is presented in Supplementary Fig. 2B

Forty-nine tissue samples positive for nematode larvae were subjected to PCR for identification targeting the ITS-1 and ITS-2 regions, which are specific for *T. canis* and *T. cati*. Amplification of the target fragments revealed that 21 chickens are positive for *Toxocara* species. Under the optimized cycling conditions, primer set YY1/NC2 amplified a product of the 330 bp uniquely from *T. canis* (57.1%, 12 out of 21) and primer set JW4/NC2 amplified the 600-bp product solely from *T. cati* (42.9%, 9 out of 21) (Fig. 2).

The age-wise distribution of *T. canis* larvae in subjects showed higher prevalence in younger chickens than older one (*P* < 0.05). On the other hand, the weight-wise distribution of subjects revealed that chickens with lower body weight were highly infected with *Toxocara* species compared to that of higher body weight chickens. This was statistically significant (*P* < 0.05) (Table 2).

**Table 2:** Species wise prevalence of toxocariasis in free-range broiler chickens by molecular method (PCR)

Parameter	Species	Infected	None-infected	***P-***value
		No. (Mean ± SD)	No. (Mean ± SD)	
Age	*T. canis*	12 (9.25 ± 5.1)	188 (16.5 ± 8.9)	0.002
	*T. cati*	9 (11.88 ± 7.9)	191 (16.3 ± 8.4)	0.074
Weight	*T. canis*	12 (1254.2 ± 759.3)	188 (1771.5 ± 794.0)	0.027
	*T. cati*	9 (1311.1 ± 760.7)	191 (1760.7 ± 797.6)	0.123

## Discussion

Consumption of raw or undercooked meat, especially chicken meat or giblets containing the larvae, is a significant route for transmission of *Toxocara* species [7, 12]. In Iran, the percentage of *Toxocara* seropositivity in humans varies from 0 to 64% and in dogs and cats as definitive hosts from 3 to 31% [19]. In some areas of northwestern Iran, the prevalence of anti-*Toxocara* IgG has been approximately 6.9% in dog and cat owners [20] and 4.5% in children with allergic manifestations [21]. However, there is no information about the prevalence of *Toxocara* in free-range broiler chickens in Iran with regard to housing systems on farms. In addition, no research has been conducted on the molecular prevalence of *Toxocara* in chicken tissues that is sold in traditional Iranian markets.

In the present study, 16.5% (33/200) of the free-range broiler chickens were positive for *Toxocara* spp. by conventional microscopic examination for the nematode larvae. Zibaei et al. [12] have recorded a somewhat lower (15.2%) prevalence than the present study, which might be attributed to the sample size, organs and target population. The high proportion of the infection found in the present study may be related to exposure of the free-range chickens to the natural environment, which is known to increase the probability of *Toxocara* infection [12]. This risk is associated with a higher probability of ingesting infective eggs from contaminated soil [22–24]. Dogs and cats may roam freely in hay and farms. The high percentage of infected poultry detected in farms may be a consequence of insufficient housing protection with low hygiene standards for chickens, which may lead to ingestion of embryonated eggs excreted by dogs and cats or ingestion of invertebrate paratenic hosts of *Toxocara* [25].

Larvae were isolated from the digested tissues by microscopy observation and subjected to conventional PCR using ITS1 and ITS2 regions for identification of *Toxocara* species. As a result, 21 chickens were confirmed positive for *Toxocara* species (10.5%, 21/200) with relative frequency of 57.1% for *T. canis* and 42.9% for *T. cati*. Similarly, Zibaei et al. [12] isolated *Toxocara* larvae from tissue samples of 33 broiler chickens from Lorestan Province of Iran and identified the *Toxocara* species by PCR. The findings of the study showed that, 5 (83.3%) were determined as *T. canis* and 1 (16.7%) as *T. cati*. In a recent study by Okada et al. [13], the liver, breast meat and thigh meats of culled chickens from a commercial layer farm were investigated by microscopy and PCR. *T. cati* and *T. tanuki* larvae were detected from 2 out of 50 chickens.

Many researchers have investigated PCR assay for differentiation of *Toxocara* species, which helps in molecular epidemiological investigations and to understand the species wise distribution of the parasite [26–28].

In the current survey, the most infected organs were gizzard and liver. This is in accordance with a similar study conducted by Taira et al. [7], who reported that the liver were infected with *T. cati* larvae during the early migration stage. Some studies reported that the gastro-intestinal tract of chickens including, the crop, gizzard, duodenum, large intestine and cloaca, has a higher chance of infection for *Toxocara* larvae than other organs [4, 9].

One of the main findings was a significant difference between recovered larvae in infected and non-infected chickens regarding age and body weight. The results indicated that the viscera of young and underweighted chickens are more affected by larvae. Although not much data has been published about the weight and age of chickens naturally infected with *Toxocara*, in several experimental studies, infection of younger chickens indicated the early accumulation of larvae in the internal organs based on the larval migration pattern [4, 7, 9]. Taira et al. [7] showed that although there was no decrease in total larval recovery over time in chickens experimentally infected with *T. cati* eggs, the migration pattern began in the liver at 1 day post-infection, migrating to the muscles at 29 days post-infection. Finally, 99.6% of larvae were recovered from the same organ 176 days post-infection. Further studies are needed to elucidate whether larval recovery in younger, naturally infected chickens is related to the larval migration pattern or is due to their high susceptibility.

## Limitations

The current study faced limitations such as resource constraints and lack of access to sequencing tests. Molecular testing with high sensitivity/specificity has been done to overcome some limitations. It is recommended to examine more tissues and organs of the samples and analyze them with molecular-based methods. Furthermore, the migration pattern of *Toxocara* species larvae on carcasses and brain will be studied in the future studies.

## Conclusion

The current study showed that *T. canis* and *T. cati* can infect paratenic hosts and molecular approaches such as conventional PCR are necessary to identify specific species. On the other hand, the identification of *Toxocara* species larvae in free-range broiler chickens indicate that chickens can be an important paratenic host for humans. Consumption of farm-raised chicken viscera, especially gizzards, liver and heart, can be associated with a high risk of human infection. Finally that this study will help to improve the detection of *Toxocara* species in animals for consumption as poultry.

## Supplementary Information

**Additional file 1 **Supplementary Fig. 2. Full-length gel images of PCR assay. Analysis of PCR products of *Toxocara* species larvae by electrophoresis on 1.5% agarose gels. **(A)** Lane C+: Positive control with standard DNA, lane M: 100-bp DNA ladder, lane C-: negative control without DNA, lanes 1-7: infected tissues. The 330-bp fragments are specific for *Toxocara canis* (lanes 1, 3, 5, and 6). **(B)** The figure shows tissues infected with *Toxocara cati*. 100-bp molecular size marker (M), positive control (C+) with standard DNA, and negative control (C-). Amplification of 600-bp *Toxocara* DNA in tissue samples from free-range broiler chickens (lanes 2, 5, 6, 8, 10-12, and 16).

**Additional file 2.** STROBE Statement Checklist of items that should be included in reports of cross-sectional studies.

## Data Availability

All the data generated or analyzed in this study are included in this published article. The datasets used and/or analyzed during the current study are available from the corresponding author on reasonable request.

## Abbreviations

DW: Distilled water
HCl: Hydrochloric acid
ITS: Internal transcribed spacer
PCR: Polymerase chain reaction
SPSS: Statistical package for the social sciences
*T. canis*: *Toxocara canis*
*T. cati*: *Toxocara cati*
