# Supplementary material for: Detection of Toxocara species larvae in four Iranian free-range broiler farms
Source: BMC Vet Res. 2022 Nov 21;18:413. doi: 10.1186/s12917-022-03516-w (PMC9680124; doi:10.1186/s12917-022-03516-w)
Supplement: Supplementary file 1 — Additional file 1 Supplementary Fig. 2. Full-length gel images of PCR assay. Analysis of PCR products of Toxocara species larvae by electrophoresis on 1.5% agarose gels. (A) Lane C+: Positive control with standard DNA, lane M: 100-bp DNA ladder, lane C-: negative control without DNA, lanes 1-7: infected tissues. The 330-bp fragments are specific for Toxocara canis (lanes 1, 3, 5, and 6). (B) The figure shows tissues infected with Toxocara cati. 100-bp molecular size marker (M), positive control (C+) with standard DNA, and negative control (C-). Amplification of 600-bp Toxocara DNA in tissue samples from free-range broiler chickens (lanes 2, 5, 6, 8, 10-12, and 16). [file 12917_2022_3516_MOESM1_ESM.doc]

**
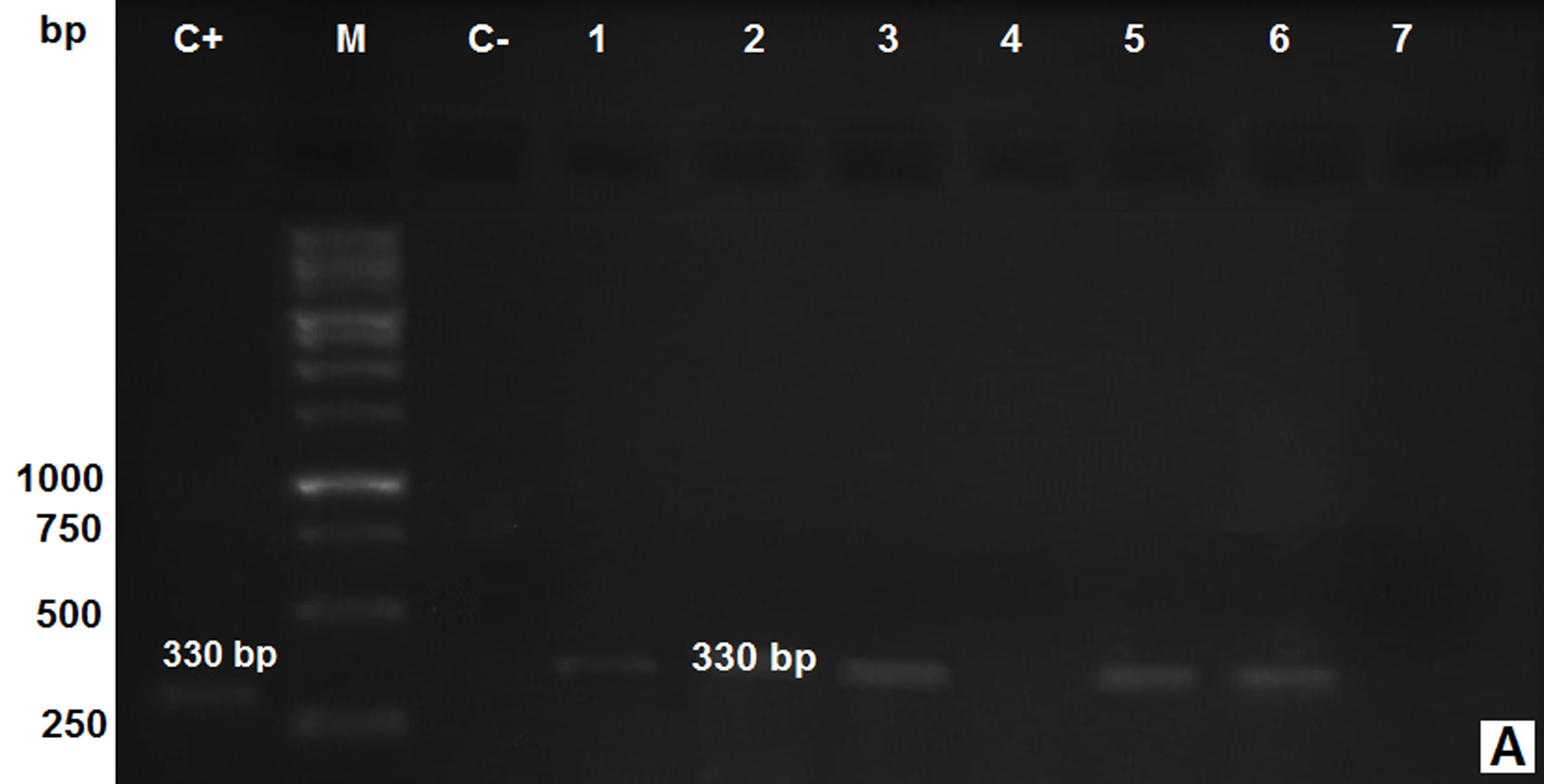
**

**
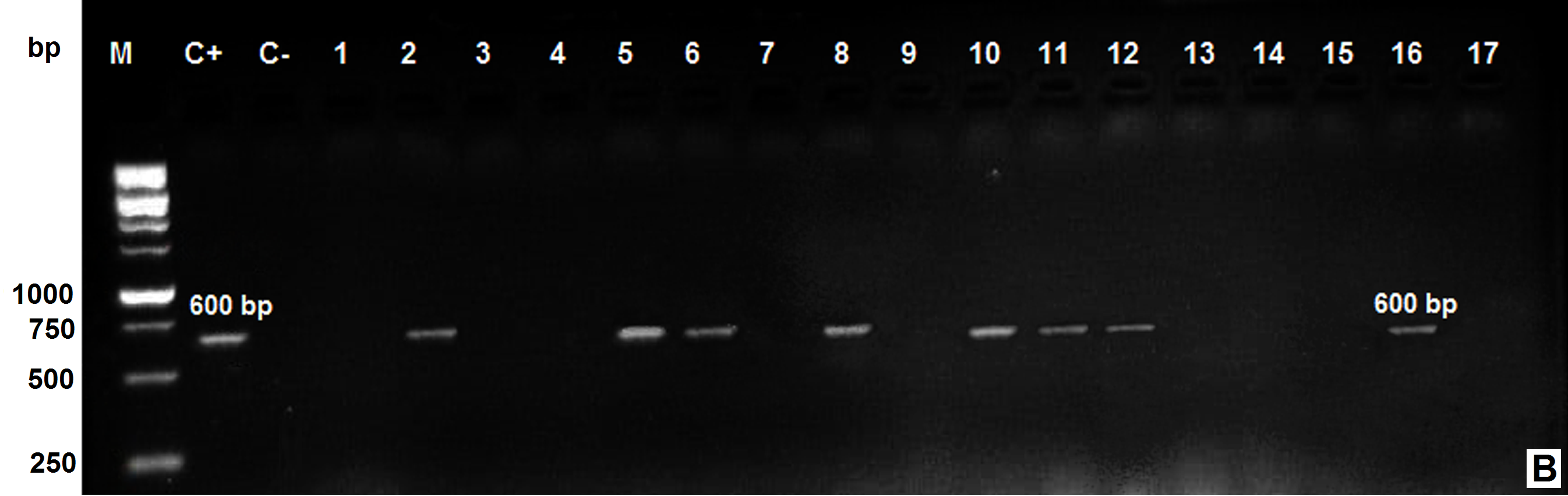
**

**Additional file 1**

Supplementary Fig. 2. Full-length gel images of PCR assay.

Analysis of PCR products of *Toxocara* species larvae by electrophoresis on 1.5% agarose gels. **(A)** Lane C+: Positive control with standard DNA, lane M: 100-bp DNA ladder, lane C-: negative control without DNA, lanes 1-7: infected tissues. The 330-bp fragments are specific for *Toxocara canis* (lanes 1, 3, 5, and 6). **(B)** The figure shows tissues infected with *Toxocara cati*. 100-bp molecular size marker (M), positive control (C+) with standard DNA, and negative control (C-). Amplification of 600-bp *Toxocara* DNA in tissue samples from free-range broiler chickens (lanes 2, 5, 6, 8, 10-12, and 16).

**Additional file 2**

STROBE Statement Checklist of items that should be included in reports of cross-sectional studies.
