# Supplementary material for: Detection of Toxocara species larvae in four Iranian free-range broiler farms
Source: BMC Vet Res. 2022 Nov 21;18:413. doi: 10.1186/s12917-022-03516-w (PMC9680124; doi:10.1186/s12917-022-03516-w)
Supplement: Supplementary file 2 — Additional file 2. STROBE Statement Checklist of items that should be included in reports of cross-sectional studies. [file 12917_2022_3516_MOESM2_ESM.doc]

STROBE Statement—Checklist of items that should be included in reports of ***cross-sectional studies***

|  | Item No | Recommendation |
| --- | --- | --- |
| **Title and abstract** | 1 | (*a*) Detection of *Toxocara* species larvae in four Iranian free-range broiler farms |
|  |  | (*b*) **Background:** The epidemiology of *Toxocara canis* and *Toxocara cati* in food animals, associated products, and their zoonotic potential are poorly understood. A cross sectional study was designed to determine the prevalence of *Toxocara* spp. larvae from free-range broiler chickens in traditional farms using conventional techniques and molecular method. Eight-hundred tissue samples including liver, gizzard, lungs and heart were collected from 200 chickens belonging to different regions of Zanjan Province, Iran and were processed by conventional and molecular methods.  **Results:** Out of 800 chicken tissues, 49 samples (6.1%) were positive for nematode larvae. Polymerase chain reaction was performed to identify species-specific of *Toxocara* larvae. The findings showed that 10.5% (21 out of 200) chickens were infected with *Toxocara* species, so that 57.1% (12 out of 21) of the samples were positive for *Toxocara canis* and 42.9% (9 out of 21) of the samples were positive for *Toxocara cati*.  **Conclusion:** Considering the significant contamination/infection of free-range broiler chickens with *Toxocara* larvae, the consumption of chicken meat and viscera, especially liver and gizzards, can play an important role in the transmission of infection to humans. Prevention and control measures focused on regular deworming of dogs and cats, increasing public awareness of *Toxocara* infection are recommended. |
| Introduction | | |
| Background/rationale | 2 | The dog and cat roundworms, *T. canis* and *T. cati* respectively, are a public health concern in Asia, America, and parts of Europe [1]. The consumption of raw or undercooked viscera or meat of economic animals is a potential risk factor for *Toxocara* infection [2]. The infective larvae are released in the small intestine and subsequently penetrate the intestinal wall, enter the circulation and migrate to various organs, where they provoke inflammatory reactions and symptoms. Based on the organs infected, it is clinically classified into visceral larva migrans, ocular toxocariasis, neural larva migrans, and hidden and/or covert toxocariasis [3]. Known paratenic hosts of *Toxocara* include chicken, cattle, pigs, rodents and birds. Larvae can move into these tissues and survive for a long time [4, 5]. It is thought that the larvae of *Toxocara* species in chicken tissue (gizzard, lungs, kidneys, liver, and heart) are the source of infection for humans [6]. The consumption of raw or undercooked viscera or meat of economic animals is a potential risk factor for *Toxocara* infection [7]. It has been studied that in chicken, most *T. canis* larvae migrate to the liver and remain there [8-10], while most of the *T. cati* larvae migrate to and remain in the muscles [4, 11].  Owing to difficulties with conventional protocols for diagnosis, researchers utilize DNA detection methods because of its rapidity and reliability. Polymerase chain reaction was used to identify and confirm helminth parasites by various researchers worldwide to target specific genes [12-14]. Molecular tools can then provide the level of discrimination that is often not achieved by microscopy alone and which is needed to distinguish between species or intraspecific variants of the relevant parasites. Molecular characterization may provide important information about potential zoonotic risk and transmission dynamics of parasites in a human community.  Molecular epidemiological studies involving animal reservoirs are crucial for determining the sources of helminthic infection in an ecological niche, inferring pathogenic characteristics of species, and developing effective control strategies. It is the most promising method for the epidemiological studies of parasitic infection such as toxocariasis. Molecular epidemiological data on *Toxocara*-infected raw meat used for human consumption in Iran is limited, and to our knowledge, this is the first study to suggest the prevalence of toxocariasis in free-range broiler chickens. |
| Objectives | 3 | Therefore, this study was conducted to investigate the prevalence and identification of *Toxocara* species larvae in four Iranian free-range broiler traditional farms in Zanjan Province in the northwest of Iran. |
| Methods | | |
| Study design | 4 | This cross-sectional study was conducted from April to October, 2021. In each agricultural zone, a traditional market for the supply of local and free range chickens was selected. In these traditional markets, free-range broiler chickens are offered and delivered to customers after butchery. The selected centres from each agricultural zone were visited once per week and 50 chickens were selected using a 1 in 4 systematic random sampling method [5]. The chicken carcasses were transferred to a parasitology laboratory for examination. Each carcass was dipped in the container of water for washing. Then, the visceral organs including the liver, lungs, gizzard, and heart were eviscerated, separately. The tissues were fragmented into small pieces of less than 5 mm^3^ with pointed forceps for pepsin digestion and PCR assay. |
| Setting | 5 | The digestion method proposed by Azizi et al. [4] and Taira et al. [9] was used with modification. |
| Participants | 6 | (*a*) Free-rang broiler chickens of poultry farms from traditional markets. |
| Variables | 7 | Not applicable |
| Data sources/ measurement | 8* | Experimental design and protocol  Two-hundred free-range broiler chickens of poultry farms r were collected  Recovery of larvae  Briefly, the fragmented tissues were put into digestive solution [1 gr pepsin (Sigma-Aldrich, USA) and 10 mL HCl 37% (Merck, Germany) in 1000 mL distilled water] under constant stirring with a magnetic stirrer and kept 2 h at 39 ℃ to recover the remaining. The sediments were filtered through system of sieves with 246-μm apertures (60-mesh) and allowed to settle for 40 min. After the supernatant was removed, sedimented liquids were poured into tubes and centrifuged for 2 min at 1,500 rpm. Each of the sediments was transferred to rectangular plastic petri dishes (Polystyrene, BIOTEST, Zist-Azmoon, Iran), and the presence of any nematode larvae was monitored under a light microscope.  DNA extraction  The DNA from the tissue samples was extracted using the Tissue Genomic DNA Extraction Mini Kit (Favorgen Biotech, Ping-Tung, Taiwan) according to the manufacturer’s instruction. In Brief, 0.25 mL of digested sample were transferred to 1.5 mL Eppendorf tube, suspended in 200 μL of lysis buffer (FATG1 Buffer) and 20 μL of Proteinase K. The solution was mixed in a vortex and incubated for up to 3 hr at 60 ºC to lyse the sample. Then, 200 µL FATG2 buffer was added to the sample, mixed thoroughly by pulse-vortexing, and incubated at 70 ºC for 10 minutes. The solution was combined with 200 μL of absolute ethanol (Merck, Germany) and it was mixed thoroughly. The mixture transferred to the FATG Mini Column (supported by the kit) and centrifuged at full speed (~18,000 x g) for 1 min, then discarded flow-through. After an additional washing with 750 μL of wash buffer, the samples were treated with 100 μL of elution buffer for 3 min at room temperature and centrifuged at full speed for 2 min to elute DNA. Finally, purified DNA concentration measured using a NanoDrop microvolume spectrophotometer (BECO, Germany).  Molecular characterization  Identification of *Toxocara* spp. isolates was performed by PCR of the ITS region and a portion of the ribosomal large subunit. The specific forward primer JW4 (5′-ACTGTCGAGGATGAGCGTGA-3′) was used, which is specific for *T. cati* and reverse primer NC2 (5′-TTAGTTTCTTTTCCTCCGCT-3′) to amplify partial internal transcribed spacer-1 (ITS-1), complete 5.8S and ITS-2 of rDNA as described by previous studies [16, 17]. The forward primer YY1 (5′-CGGTGAGCTATGCTGGTGTG -3′) which is specific for *T. canis* as previously designed and combined with reverse primer NC2 (5′-TTAGTTTCTTTTCCTCCGCT-3′) was used to amplify partial ITS-2 as well [18]. PCR reaction was performed in Super master mix-2X 12.5 μL, primers (forward and reverse) (10 pmol/ μL) 1 μL, DNA template 5 μL, DW 5.5 μL. The PCR conditions were 95°C for 5 min (primary denaturation), followed by 94°C for 45 s (denaturation), 58 °C for 35 s (annealing), and 72 °C for 35 s for 35 cycles (extension) with a final elongation step of 72 °C for 10 min.  Amplified products were separated by electrophoresis on a 1.5% agarose gel and stained with ethidium bromide with 0.5 µg/mL ethidium bromide solutions, and a 100 kbp ladder was used as DNA size marker for estimating the size of the amplicons and photographed using a gel documentation system (UV Transilluminator, QUANTUM SD4-1000, VILBER, France). |
| Bias | 9 | Describe any efforts to address potential sources of bias |
| Study size | 10 | Explain how the study size was arrived at |
| Quantitative variables | 11 | Explain how quantitative variables were handled in the analyses. If applicable, describe which groupings were chosen and why |
| Statistical methods | 12 | (*a*) Data and frequencies of *Toxocara* species were entered into SPSS 20.0 Statistics software and subjected to descriptive statistics to determine their percentages. Mann-Whitney, Kruskal-Wallis, and Chi-square (Fisher’s exact) tests were used to evaluate associations of the variables. Differences in all statistical tests were considered significant at *P* < 0.05. |
|  |  | (*b*) Describe any methods used to examine subgroups and interactions |
|  |  | (*c*) Explain how missing data were addressed |
|  |  | (*d*) If applicable, describe analytical methods taking account of sampling strategy |
|  |  | (*e*) Describe any sensitivity analyses |
| Results | | |
| Participants | 13* | (a) Free-rang broiler chickens of poultry farms from traditional markets |
|  |  | (b) Give reasons for non-participation at each stage |
|  |  | (c) Consider use of a flow diagram |
| Descriptive data | 14* | (a) Free-rang broiler chickens of poultry farms from traditional markets in Zanjan |
|  |  | (b) Two-hundred free-range broiler chickens of poultry farms r were collected |
| Outcome data | 15* | Of the 200 chickens were examined in the present study, 44 (22.0%) were male and 156 (78.0%) were female. Their age range was between 2 and 48 (16.1 ± 8.4) months. The weight of the study subjects were between 150 and 4000 (1740.5 ± 799.7) grams. Out of 800 tissue samples screened, 49 samples (6.1%) were positive for the nematode larvae by conventional microscopic examination (Figure 1). The presences of the larvae were demonstrated in the gizzard (11.5%), liver (9.5%), lungs (2.0%) and heart (1.5%), respectively.  The 49 positive tissue samples for the nematode larvae were subjected to PCR for detection by targeting ITS-1 and ITS-2 regions which are specific for *T. canis* and *T. cati*. The amplification of target fragments revealed that 21 chickens are positive for *Toxocara* species. Under the optimized cycling conditions, primer set YY1/NC2 amplified a product of 330 bp uniquely from *T. canis* (57.14%, 12 out of 21) and primer set JW4/NC2 amplified a product of 600 bp solely from *T. cati* (42.86%, 9 out of 21). The age-wise distribution of *T. canis* larvae in the subjects showed higher prevalence in younger chickens than older one (*P* ˂ 0.05). On the other hand, the weight-wise distribution of the subjects revealed that chickens with lesser weight were highly infected with *Toxocara* species compared to that of larger body weight chickens. This was statistically significant (*P* ˂ 0.05). |
| Main results | 16 | (*a*) Give unadjusted estimates and, if applicable, confounder-adjusted estimates and their precision (eg, 95% confidence interval). Make clear which confounders were adjusted for and why they were included |
|  |  | (*b*) Report category boundaries when continuous variables were categorized |
|  |  | (*c*) If relevant, consider translating estimates of relative risk into absolute risk for a meaningful time period |
| Other analyses | 17 | Report other analyses done—eg analyses of subgroups and interactions, and sensitivity analyses |
| Discussion | | |
| Key results | 18 | In the present study, 16.5% (33/200) of the free-range broiler chickens were positive for *Toxocara* spp. by conventional microscopic examination for the nematode larvae. Zibaei et al. [12] has recorded somewhat lower (15.2%) prevalence than the present study which might be attributed to the sample size, organs and target population. In fact, the high proportion of the infection were found in the present study may be related to exposure of the free-range chickens to the natural environment, which is known to increase the probability of *Toxocara* infection [12]. This risk is associated with a higher probability of ingestion of ingesting eggs from contaminated soil [22-24]. Dogs and cats may roam freely in hay and farms. The high percentage of infected poultry detected in farms may be a consequence of insufficient housing protection with low hygiene standards for chickens, which may lead to ingestion of larval eggs excreted by dogs and cats or ingestion of invertebrate paratenic hosts of *Toxocara* [25].  The tissue samples which were positive for larvae by microscopy were re-subjected to molecular method to identification of *Toxocara* species by targeting ITS1 and ITS2 regions. As a result, 21 chickens were confirmed positive for *Toxocara* species (10.5%, 21/200) with relative frequency of 57.1% for *T. canis* and 42.7% for *T. cati*. |
| Limitations | 19 | The present study faced limitations such as resource constraints and lack of access to sequencing tests. Molecular testing with high sensitivity/specificity has been done to overcome some limitations. |
| Interpretation | 20 | Give a cautious overall interpretation of results considering objectives, limitations, multiplicity of analyses, results from similar studies, and other relevant evidence |
| Generalisability | 21 | Discuss the generalisability (external validity) of the study results |
| Other information | | |
| Funding | 22 | The authors state that they have not received any funding or grants to write and publish the results of this study. |

*Give information separately for exposed and unexposed groups.

**Note:** An Explanation and Elaboration article discusses each checklist item and gives methodological background and published examples of transparent reporting. The STROBE checklist is best used in conjunction with this article (freely available on the Web sites of PLoS Medicine at http://www.plosmedicine.org/, Annals of Internal Medicine at http://www.annals.org/, and Epidemiology at http://www.epidem.com/). Information on the STROBE Initiative is available at www.strobe-statement.org.
